# Drugging Challenging Cancer Targets Using Fragment-Based Methods

**DOI:** 10.1021/acs.chemrev.4c00892

**Published:** 2025-03-05

**Authors:** Stephen W. Fesik

**Affiliations:** Department of Biochemistry, Chemistry, and Pharmacology, Vanderbilt University, Nashville, Tennessee 37235 United States

## Abstract

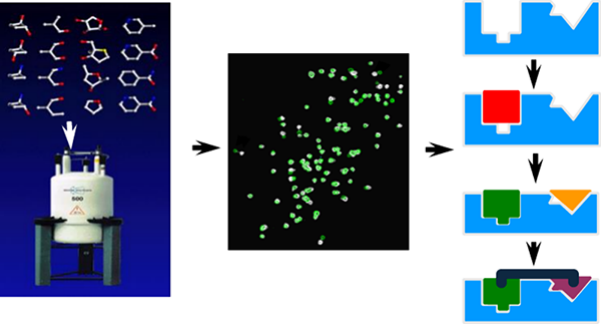

There are many highly validated cancer targets that are difficult or impossible to drug due to the absence of suitable pockets that can bind small molecules. Fragment-based methods have been shown to be a useful approach for identifying ligands to proteins that were previously thought to be undruggable. In this review, I will give an overview of fragment-based ligand discovery and provide examples from our own work on how fragment-based methods were used to discover high affinity ligands for challenging cancer drug targets.

## 1 Introduction

**Figure 1:**
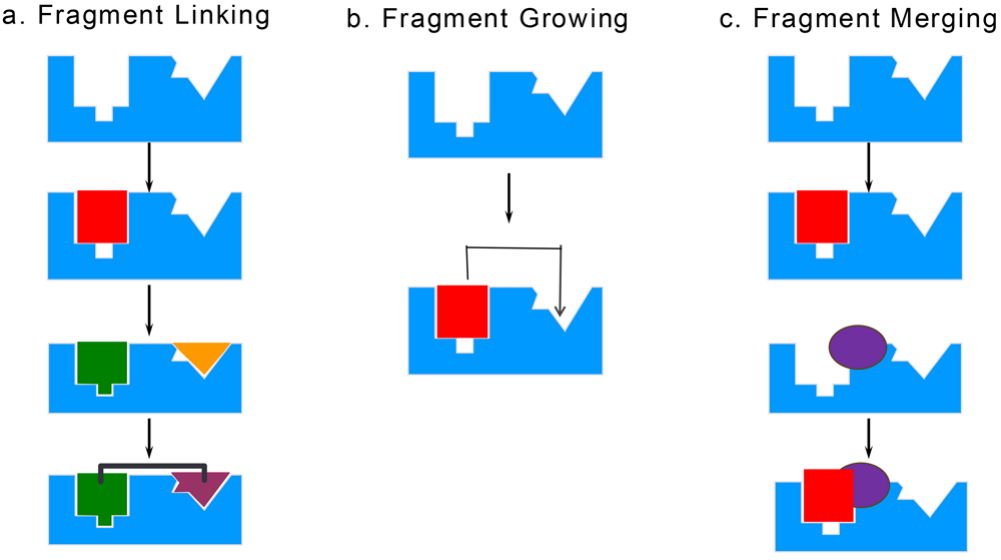
Optimization of fragment-based hits by (a) linking, (b) growing, or (c) merging.

In 1996, we described a method for identifying small organic molecules (fragments) that bind to proteins using NMR that could serve as useful starting points for discovering drugs.^1^ We called the method SAR by NMR (Structure Activity Relationships by Nuclear Magnetic Resonance). This was the first demonstration of fragment-based methods for identifying high affinity ligands.^1,2^ Using this method, small molecules (MW < 300) that bind to a protein are identified by the chemical shift changes observed by NMR. Theoretically, screening small molecules vs larger molecules can better cover chemical space^3^ with greater ligand efficiency.^4^ Although fewer interactions with the protein are observed with small molecules, these interactions are generally of higher quality. Also, because small molecules are less complex, they do not contain extra functional groups which can interfere with binding to the protein.^5^ However, due to the paucity of interactions that stabilize complex formation, the fragments are expected to bind only weakly to proteins, and therefore a robust method is needed for reliably detecting weakly bound ligands. Although other approaches have been used such as surface plasmon resonance (SPR), X-ray crystallography, ligand-detected NMR,^6^ observing the isotopically labeled protein NMR signals is ideally suited for this purpose because there is no background interference. This advantage reduces false positives and false negatives.^6^ Fragment binding is detected from the chemical shift changes induced by the ligand, and if the protein chemical shifts have been assigned, then the location of the binding site can be determined. In addition, by following the shift changes as a function of the concentration of the ligand, binding constants can be measured. Another advantage is that analogues of such small molecules hits can be readily purchased or easily synthesized to explore the structure activity relationships (SAR) and obtain molecules with improved binding affinity. To further improve the binding affinity, a linked fragment approach can be used in which a second site screen is performed in the presence of a first ligand (Figure 1a). To determine how to link the two ligands together to greatly improve the affinity, the structure of the ternary complex is obtained. Theoretically, Jencks^7^ postulated that the binding affinity of a linked ligand is equal to the binding affinity of one fragment times the binding affinity of the second. Additional gains are obtained due to entropic factors. Therefore, ligands that bind with mM affinities, if linked together properly, can produce molecules with submicromolar affinities as demonstrated in 1996 by the discovery of high affinity (nM) ligands for FKBP^1^ and in 1997 by the discovery of high affinity (nM) inhibitors of the matrix metalloproteinase stromelysin^8,9^ by linking together two weakly bound fragments. Other useful approaches for improving weakly bound fragment hits have emerged such as fragment growing (Figure 1b) and fragment merging (Figure 1c), which have been successfully used to discover high affinity ligands for many proteins.^10−12^

Since our first application of fragment screening, we have used this approach to discover small molecules that bind to several proteins, including human papillomavirus E2 protein,^13^ Erm methyl transferase,^14^ the SH2 domain of lck,^15^ urokinase,^16^ adenosine kinase,^17^ protein tyrosine phosphatase 1B,^18^ XIAP,^19^ Bcl-xL,^20,21^ Bcl-2,^22,23^ Mcl-1,^24−26^ KRAS,^27,28^ replication protein A,^29^ the bromodomain of ATAD2,^30^ WDR5-WIN-Site,^31^ WDR5-WBM-Site,^32,33^ SOS,^34−36^ PD-L1,^37^ and Tim3.^38^ In the last 25 years, fragment-based screening has been widely accepted and applied in both academia and industry by us and others. Fragment-based screening has become one of the preferred methods used for identifying hits that bind to proteins that serve as starting points for drug discovery. It has led to the discovery of 7 drugs^23,39−43^ and over 60 compounds in clinical trials.

One of the unique advantages of the fragment-based approach to drug discovery is the ability to obtain high affinity ligands for challenging protein targets that lack suitable pockets for binding to small molecules.^44^ This is particularly important for highly validated protein targets that exert their activity through protein–protein or protein–DNA interactions.^45−47^ One of the first successful applications of fragment-based methods for targeting a protein–protein interaction is the discovery of inhibitors for the Bcl-2 family of proteins that resulted in an approved drug, venetoclax, for the treatment of chronic lymphocytic leukemia (CLL) and acute myeloid leukemia (AML) as described in the next section.

## 2 Bcl-2 Family of Proteins

**Figure 2:**
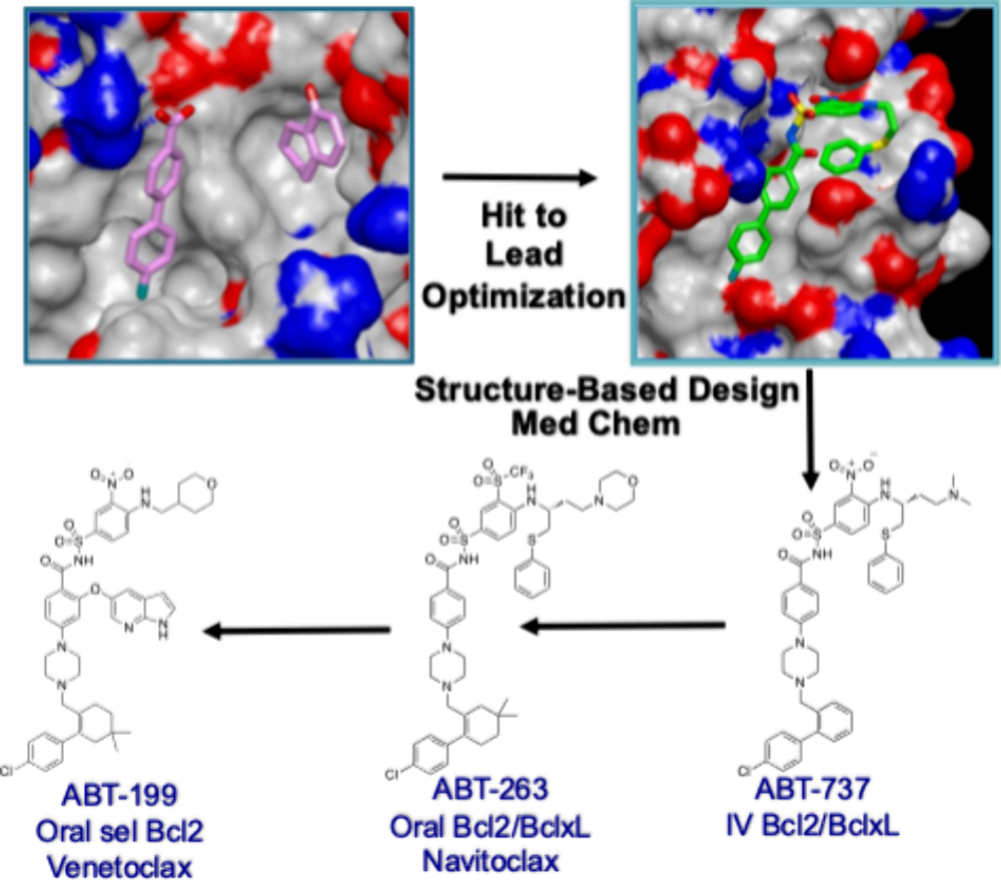
Two fragments were identified that bind nearby in Bcl-xL (PDB 1YSG). They were linked together and optimized to generate a lead (PDB 1YSI) that was subsequently further improved by structure-based design and extensive medicinal chemistry to produce ABT-737.^20^ To obtain an orally active inhibitor of Bcl-2, Bcl-xL, and Bcl-w, ABT-263 (Navitoclax) was discovered that entered clinical trials.^53^ To avoid the toxicities observed with ABT-263, a Bcl-2 selective inhibitor (ABT-199, Venetoclax)^23^ was discovered that was approved for CLL and AML. Adapted from figure 1 with permission from ref (20). Copyright 2005 Nature.

The Bcl-2 family of proteins regulate apoptosis through interactions of the antiapoptotic and pro-apoptotic members of the same family. Since protein–protein interactions were thought to be difficult or impossible to drug, this class of proteins were not considered to be favorable drug targets. However, based on the hydrophobic groove observed in the structures of Bcl-xL^48^ and the Bcl-xL/Bak peptide complex,^49^ it was hypothesized that it might be possible to discover a small molecule that could bind tightly to Bcl-xL and displace Bak and other pro-apoptotic members of the Bcl-2 family to cause the death of cancer cells. Although a high throughput screen of the Abbott compound library did not yield any useful hits that could be used as starting points for drug discovery, fragment-based screening was successful in finding small molecules that bind to a pocket in Bcl-xL and to a second nearby site (Figure 2).^20^ Structural information obtained by NMR on how these small fragments bind to Bcl-xL was then used to guide how to link them together which produced Bcl-xL inhibitors that were optimized to produce more potent analogues using structure-based design.^50−52^ After several years of effort, a subnanomolar inhibitor of Bcl-2, Bcl-w, and Bcl-xL (ABT-737) was obtained that induced the regression of solid tumors (Figure 2).^20^ This was one of the first demonstrations of effectively targeting a protein–protein interaction lending hope that other such targets may be tractable using fragment-based methods and structure-based design.

ABT-737 was not perfect, however, as it lacked oral bioavailability, These problems with ABT-737 were fixed by the discovery of ABT-263 (Navitoclax) an orally bioavailable analogue of ABT-737 (Figure 2) that could also potently inhibit Bcl-2, Bcl-xL, and Bcl-w.^53^ Navitoclax entered clinical trials, and the dose limiting toxicities observed was thrombocytopenia and cardiotoxicity. Since these toxicities were found to be due to the inhibition of Bcl-2, a selective Bcl-2 inhibitor was sought. Several approaches were attempted, including fragment screening using a loop swapped mutant of Bcl-2.^22^ Ultimately, success was achieved by determining the X-ray structure of Bcl-xL complexed with an analogue of ABT-263 that lacked the S-phenyl group. The structure revealed that a tryptophan from an adjacent Bcl-2 protein in the crystal structure occupied the same space as the S-phenyl of ABT-263. Based on this structural information, a Bcl-2 inhibitor was designed and synthesized that contained an ether linked indole which mimicked the tryptophan and formed a pi stacking interaction as predicted from the structure. This compound was further optimized by preparing an azaindole analogue to produce ABT199, a Bcl-2 selective subnanomolar inhibitor of Bcl-2 that exhibited 3 orders of magnitude less binding to Bcl-xL(Figure 2).^23^ As expected, ABT199 did not cause a reduction in platelets or cardiotoxicity and was very active against several cancers that were dependent on Bcl-2 for survival. Based on the remarkable efficacy and lack of significant toxicities, ABT 199 (Venetoclax) was approved for patients with chronic lymphocytic leukemia^54^ and subsequently AML.^55^ Venetoclax represents one of the seven drugs discovered by fragment-based screening.

MCL-1 is a also a member of the antiapoptotic class of Bcl-2 family of proteins. It is a well validated cancer target that is overexpressed in many cancers and allows cancer cells to avoid apoptosis. However, like other antiapoptotic members of the Bcl-2 family, MCL-1 is considered difficult to drug.

To identify starting points for discovering MCL-1 inhibitors, a fragment-based screen was conducted in which two hits were identified that bound to different sites on the protein. In this case, a fragment merging strategy was employed to obtain a more potent MCL-1 inhibitor.^24^ After extensive optimization,^56−59^ we discovered highly selective, picomolar MCL-1 inhibitors with enhanced cellular potency, drug-like PK properties, and potent antitumor activities in heme malignancies and lung xenograft models. Potent Mcl-1 inhibitors were also discovered by others that entered clinical trials.^60−63^ However, due to the cardiotoxicity observed in patients, their development has slowed or halted. To retain their anticancer activity but avoid the cardiotoxic side effects, Mcl-1 inhibitors have been designed with very short half-lives (e.g., BRD-810) which can induce irreversible apoptosis in tumor cells while eliminating cardiotoxicity.^64^

## 3 KRAS

KRAS is a GTPase that is heavily mutated in many cancers, including 86–96% of pancreatic cancers, 40–54% of colorectal cancers, and 27–39% of lung cancers. Activating mutations in KRAS increase signaling in several important cellular pathways and are the most common oncogenic drivers in human cancer. Thus, KRAS is a highly validated cancer target. However, despite many attempts to target KRAS over decades, it was long thought impossible to drug. Using NMR-based fragment screens, we identified small molecules that bind to KRAS in the active GTP- and inactive GDP-bound forms.^27^ Subsequent optimization of the fragment hits that bind to KRAS-GTP using structure-based design led to a compound that binds to the switch I/II site with nanomolar affinity, inhibits all GEF, GAP, and effector interactions with KRAS, and displays an antiproliferative effect in KRAS mutant cells.^28^

Shokat and co-workers^40^ used a tethering strategy to identify compounds that covalently bind to G12C KRAS. These compounds bound to the switch II pocket on KRAS. This ground breaking study led to the discovery of several G12C inhibitors that are approved for the treatment of lung cancers that harbor a G12C KRAS mutant.^65^

**Figure 3:**
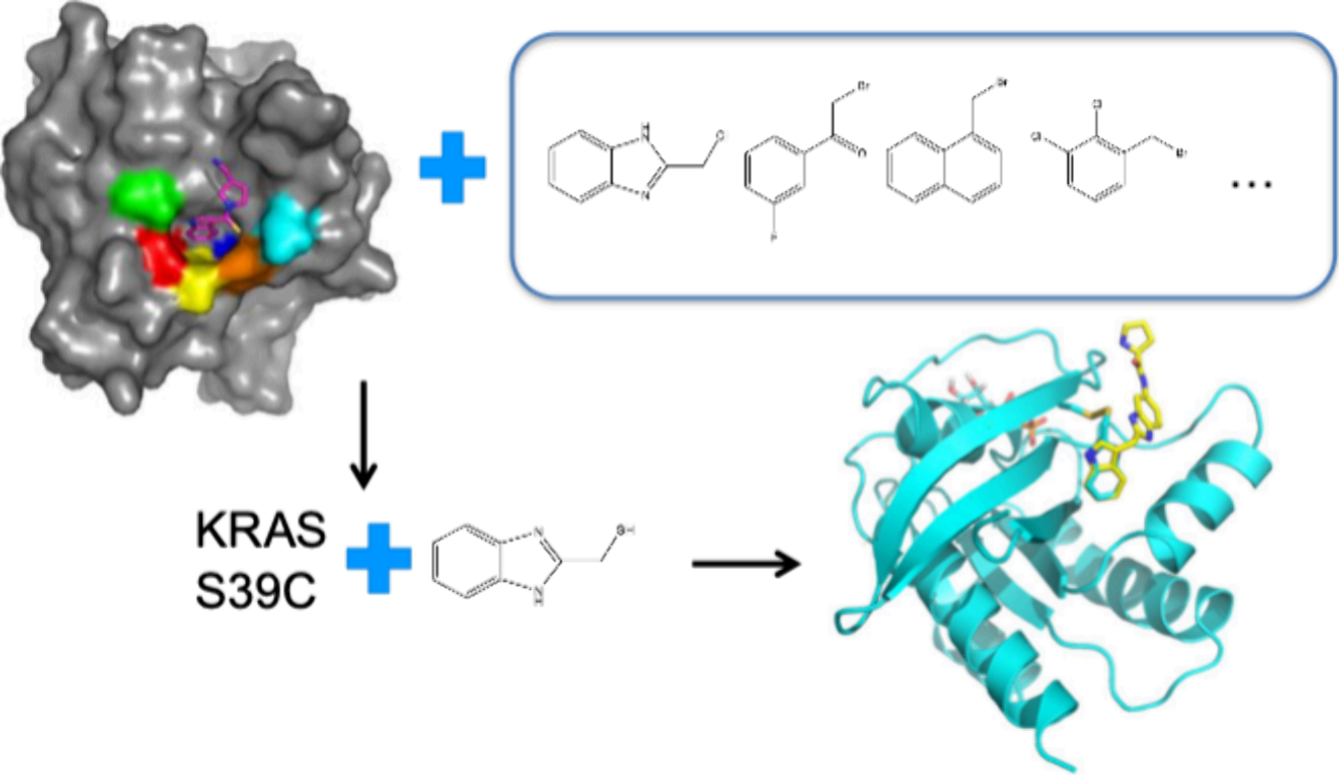
To fix the first site fragment hit into the switch I/II site of KRAS, six cysteine mutants were prepared as shown in different colors. These mutant proteins were reacted with a library of electrophiles to identify a compound and a cysteine mutant that would form a covalent interaction without altering the KRAS structure as evidenced by the overlay of a bound noncovalent KRAS inhibitor (yellow) superimposed with the covalently bound molecule (blue) (PDB 4PZZ).^66^ Adapted with permission from ref (66). Copyright 2024 Springer Science.

**Figure 4:**
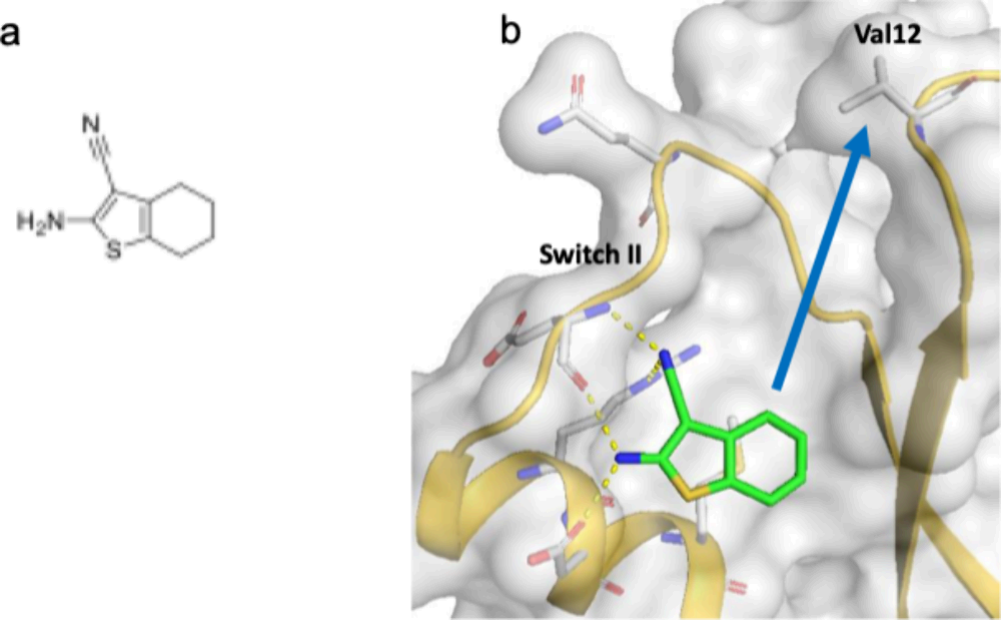
(a) A small molecule hit identified in an NMR-based second-site screen. (b) The X-ray structure of G12V KRAS bound to the fragment hit (PDB 7U8H). The compound binds to the switch II pocket of KRAS with a direct path to the amino acid residues at the 12 position (arrow) where the cancer causing KRAS mutants are located.^67^ Adapted with permission from ref (67). Copyright 2022 American Chemical Society.

**Figure 5:**
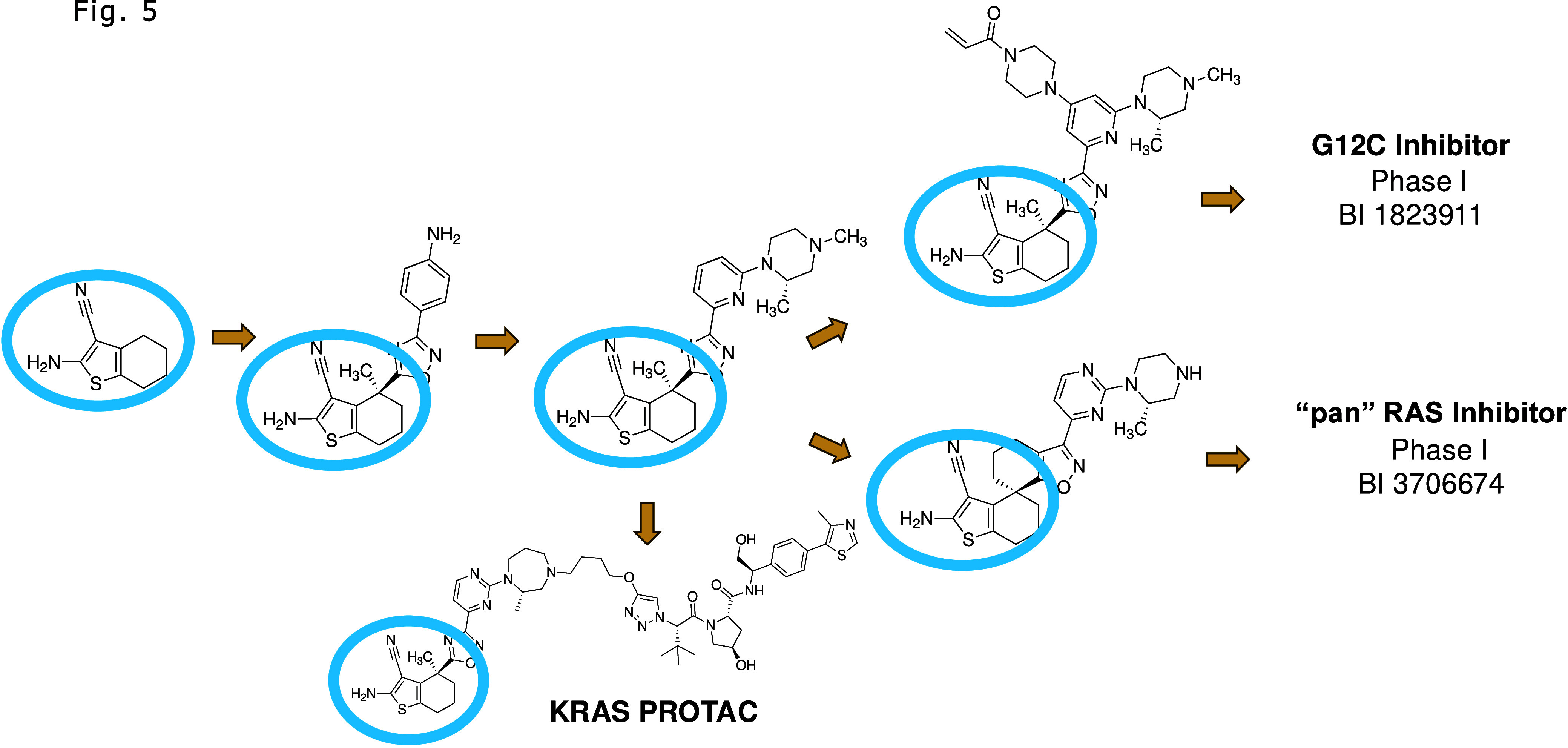
Discovery of a G12C KRAS inhibitor,^67^ a pan KRAS inhibitor,^68^ and a KRAS/degrader^69^ based on the second site fragment hit. All of the Boehringer Ingelheim KRAS inhibitors/degraders contain the second site hit as indicated by the blue ovals.

We also identified compounds that bind to the switch II pocket on KRAS using a different approach. We conducted a second site screen of KRAS-GDP to identify compounds that could be linked or merged to the first site hits that bind to switch I/II. However, the molecules simply displaced the first site ligand in the screen. We therefore developed a strategy to hold the first site ligand in place by introducing cysteine mutants near the switch I/II site and covalently attaching molecules to these cysteines (Figure 3).^66^ Using S39C KRAS with a covalently bound switch I/II blocker, a second site screen was performed in which 20 hits were identified that bound to KRAS at a second site. We obtained X-ray crystal structures that showed these hits unexpectedly bind to the switch II site (Figure 4), the same site found by Shokat and co-workers. Optimization using structure-based design with Boehringer Ingelheim (BI) led to BI 1823911,^67^ a G12C inhibitor in phase I, a pan KRAS inhibitor (BI 3706674)^68^ which has recently entered phase I, a pan-RAS degrader,^69^ and mutant selective KRAS inhibitors that are currently in preclinical studies. Interestingly, the reported KRAS inhibitors/degraders under development from BI all contain the fragment that we identified from the second site screen (Figure 5).

## 4 MYC

**Figure 6:**
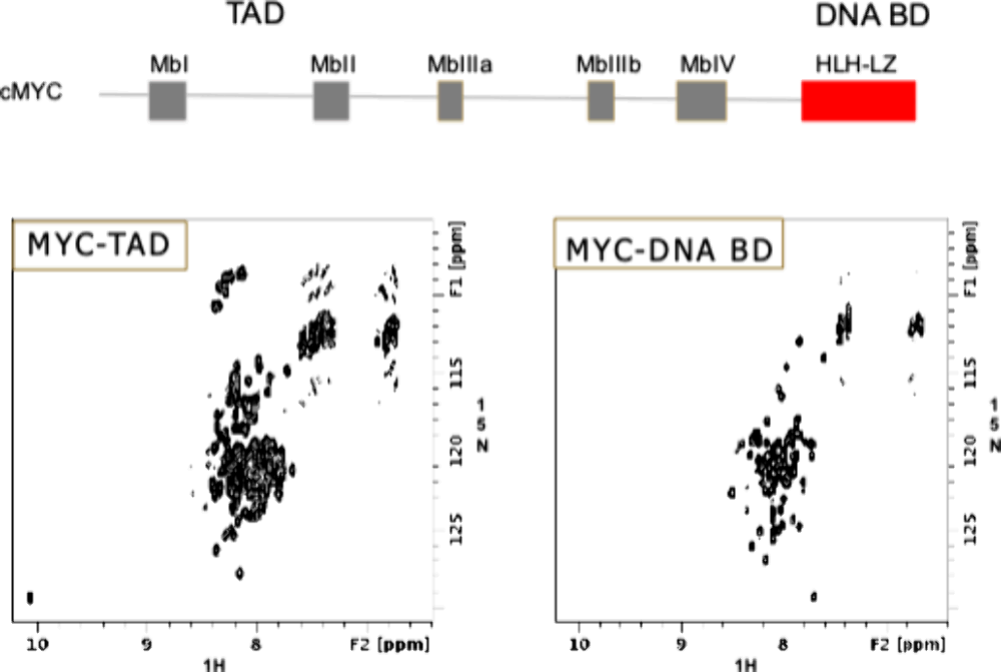
^1^H/^15^N HSQC spectra of the transactivation domain (TAD) and DNA binding domain of MYC, indicating that both regions of MYC are disordered in solution.

MYC is a transcription factor that is overexpressed in most cancers. MYC is composed of an N-terminal transactivation domain, a C-terminal basic-helix–loop–helix leucine zipper DNA binding domain, and a central region (Figure 6). It forms a heterodimer with MAX, binds to E-box DNA, and drives the expression of genes required for cell growth, proliferation, metabolism, genome instability, and apoptosis. Like KRAS, MYC is a highly validated target but is thought to be difficult or impossible to drug, but unlike KRAS, no small molecules that directly target MYC have been approved to date.

Our first attempt to drug MYC used an NMR-based fragment screen of the transactivation domain and the DNA binding domain of MYC, two regions of the protein required for activity. No confirmed hits were found for either of these proteins. This outcome was not surprising, as MYC, in the absence of MAX, is intrinsically disordered in solution as evidenced by the poor chemical shift dispersion observed in our NMR spectra (Figure 6).

**Figure 7:**
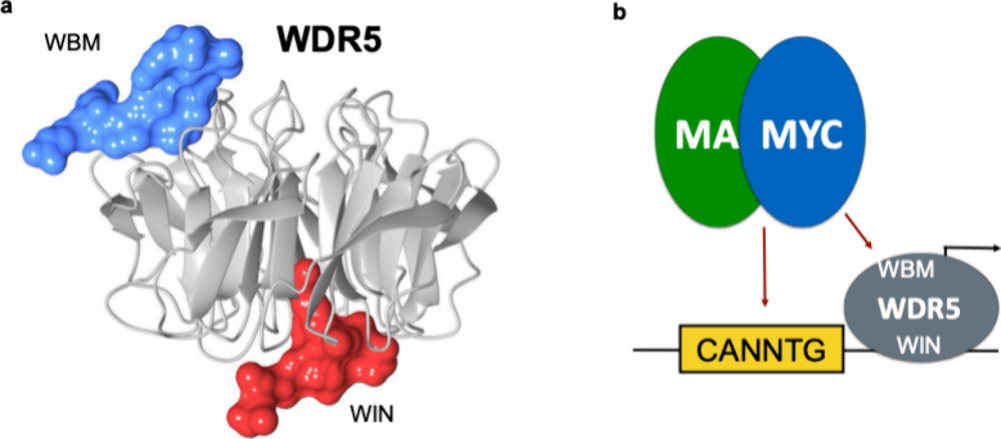
(a) Ribbon plot of WDR5 showing the location of the WBM (where MYC binds, blue) and the WIN site (where MLL1 binds, red). (b) Model for the function of MYC. The MYC/MAX dimer binds to E-box DNA while the central portion of MYC (MYC box iiib) binds to the WBM site of WDR5. The WIN site localizes WDR5 (and MYC) to chromatin.

**Figure 8:**
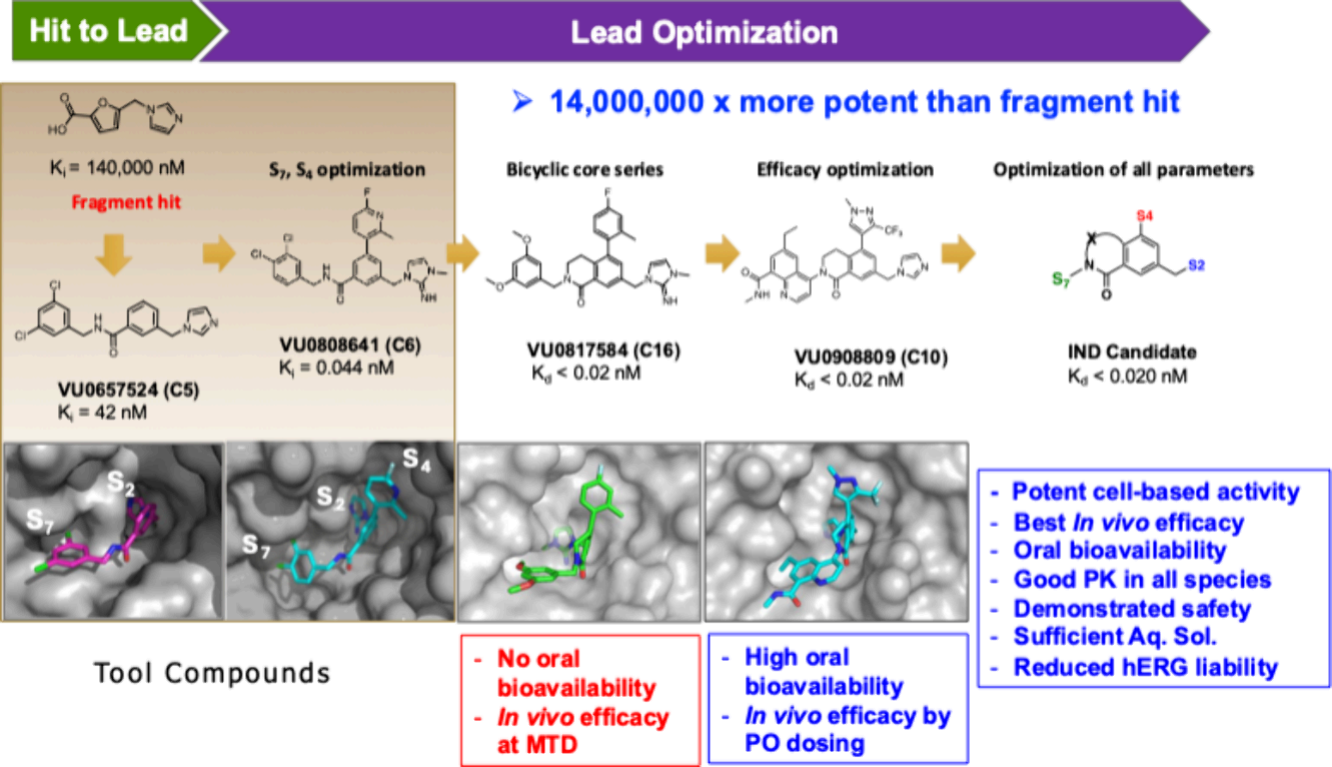
Discovery of WIN site WDR5 inhibitors starting with a fragment hit to generate tool compounds with sufficient potency for hit validation studies (VU0657524, PDB 6DYA; VU0808641, PDB 6E23). These compounds were then further optimized for their potency and drug-like properties (VU0817584, PDB 6UCS; VU0908809, PDB 8E9F) to yield a WDR5 inhibitor with a 14 million fold improvement in binding affinity that was suitable for clinical trials.

Another approach for targeting MYC is to target a cofactor that might be more druggable rather than MYC itself. Using a two-hybrid and proteomic screen, Professor Bill Tansey at Vanderbilt discovered that the central portion of MYC (MYC box IIIb) binds to WDR5, and this interaction is required for MYC-driven tumorigenesis.^70^ From a fragment-based screen of WDR5, we identified hits that bound to the site where MYC interacts with WDR5 (WBM site)^32,33^ and hits that bind to the opposite end of the protein at the site where MLL1 binds to WDR5 (WIN site) (Figure 7).^31^ From these fragments, we discovered potent tool compounds that bind to the WBM and the WIN sites (Figure 8) that allowed us to study the biology of WDR5 and evaluate WDR5 as a cancer drug target. We found that WDR5 facilitates the recruitment of MYC to chromatin to control the expression of genes linked to ribosome biogenesis, a critical tumor-sustaining function of MYC.^71^ Importantly, disrupting the MYC-WDR5 interaction with chromatin promotes tumor regression *in vivo*, validating the importance of WDR5 for tumor maintenance by MYC.^72^ WIN site inhibitors act by displacing WDR5 from chromatin at ribosomal protein genes and not by changes in histone methylation as previously thought.^73,74^ These findings indicate that a WDR5 inhibitor may be useful for treating a wide range of cancers and encouraged us to further optimize the tool compounds into drugs. Upon extensive optimization (Figure 8),^75−77^ we discovered single digit picomolar WDR5 inhibitors that are orally bioavailable, efficacious *in vivo*, and safe. We have selected a candidate for IND-enabling studies and are currently working with the NCI to advance this molecule into the clinic.

## 5 Conclusions

In the last 25 years, fragment-based screening has become an important and widely used method for finding hits that bind to proteins, providing a good start to the drug discovery process. The screening of fragments containing electrophiles that bind covalently to cysteines^78^ and other amino acids^79^ of target proteins have also been described. To optimize the hits, fragment linking, fragment growing, and fragment merging are used when structures of the fragment hits bound to the target protein are known. There are now multiple success stories and examples that illustrate the power of fragment-based methods. In addition to its utility in drug discovery in general, fragment-based methods can be used to discover high affinity ligands for difficult or challenging cancer targets that are highly validated as I have illustrated here. From this work, new cancer therapies may be developed that will improve our ability to effectively treat cancer patients.
